# Randomized Controlled Trial of Motivational Enhancement Interventions to Increase Pre-Exposure Prophylaxis Use in Sexual Minority Men Who Use Stimulants

**DOI:** 10.21203/rs.3.rs-2787003/v1

**Published:** 2023-04-20

**Authors:** Leah Davis-Ewart, Christian Grov, Rachel Verhagen, Jennifer Manuel, Michael Viamonte, Samantha Dilworth, Omar Valentin, Emily M. Cherenack, Sidney Carr, Susanne Doblecki-Lewis, Inbal Nahum-Shani, Adam W. Carrico

**Affiliations:** University of Miami Miller School of Medicine; City University of New York; University of Miami; University of California, San Francisco; University of Miami Miller School of Medicine; University of Miami Miller School of Medicine; University of Miami Miller School of Medicine; University of Miami Miller School of Medicine; University of Miami Miller School of Medicine; University of Miami Miller School of Medicine; University of Michigan Institute for Social Research; University of Miami Miller School of Medicine

**Keywords:** cocaine, contingency management, methamphetamine, motivational interviewing, pre-exposure prophylaxis

## Abstract

**Background:**

Although pre-exposure prophylaxis (PrEP) could substantially mitigate HIV risk, sexual minority men (SMM) who use stimulants commonly experience difficulties with engaging in PrEP clinical services. Motivational interviewing (MI) and contingency management (CM) reduce substance use and condomless anal sex in this population, but these motivational enhancement interventions require adaptation to promote engagement along the PrEP care continuum.

**Methods:**

PRISM is a pilot sequential multiple assignment randomized trial (SMART) testing the feasibility, acceptability, and preliminary effectiveness of distinct combinations of telehealth MI and CM in 70 cisgender SMM who use stimulants that are not currently taking PrEP. A national sample was recruited via social networking applications to complete a baseline assessment and mail-in HIV testing. Those with non-reactive HIV results are randomized to receive either: 1) a 2-session MI intervention focusing on PrEP use (session 1) and concomitant stimulant use or condomless anal sex (session 2); or 2) a CM intervention with financial incentives for documented evidence of PrEP clinical evaluation by a medical provider ($50) and filling a PrEP prescription ($50). At the 3-month follow-up assessment, participants who report they have not filled a prescription for PrEP are randomized a second time to either: 1) Switch to a second-stage intervention (i.e., MI + CM or CM + MI); or 2) Continue with assessments only. Outcomes for both responders and non-responders are reassessed at a 6-month follow-up. The primary outcome is documented evidence of filling a PrEP prescription. Self-reported, secondary outcomes include PrEP clinical evaluation by a medical provider, stimulant use, and condomless anal sex. Qualitative exit interviews are conducted with a sub-group of responders and non-responders to characterize their experiences with the MI and CM interventions.

**Discussion:**

Implementation of this pilot SMART underscores the challenges in reaching SMM who use stimulants to optimize HIV prevention efforts such that approximately one in ten (104/1,060) eligible participants enrolled. However, 85% (70/82) of enrolled participants with non-reactive HIV results were randomized. Further research is needed to determine the effectiveness of telehealth MI and CM for supporting PrEP use in SMM who use stimulants.

**Trial Registration:**

This protocol was registered on clinicaltrials.gov (NCT04205487) on December 19, 2019.

## Background

Sexual minority men (SMM) continue to account for more than two-thirds of new HIV infections in the United States [1–4] and it is estimated that 70% of seroconversions in this population occur during receptive condomless anal sex (CAS) [5–7]. Consequently, expanded efforts are needed to optimize the benefits of pre-exposure prophylaxis (PrEP) among SMM at greatest risk for HIV. The Centers for Disease Control and Prevention (CDC) has estimated that one in six SMM will acquire HIV in their lifetime, including half of Black SMM and one-quarter of Latino SMM [2, 8]. Over and above these profound racial disparities, there is a resurgent epidemic of methamphetamine use among SMM that is disproportionately affecting Black and Latino SMM [9–12]. Although methamphetamine use appeared to decline somewhat after significant public health attention in the early- and mid-2000’s [13, 14], it is again on the rise [15–20]. For over two decades, methamphetamine and other stimulant use has been identified as a prominent driver of the HIV/AIDS epidemic in SMM that is associated with engagement in CAS [21, 22], altered rectal immune function [23, 24], and faster rates of HIV seroconversion [25–27]. The public health impact of stimulant use is evidenced by recent findings from a cohort of sexual and gender minorities who have sex with men where one-in-three new HIV infections were among those reporting methamphetamine use [27].

SMM who use stimulants experience substantial barriers to navigating HIV prevention services that undermine the clinical and public health benefits of PrEP. This is evidenced by findings from one study where SMM who use methamphetamine had five-fold greater odds of repeat prescription for post-exposure prophylaxis, and a three-fold greater rate of HIV seroconversion [28]. This underscores the clear benefits of supporting entry or re-entry of SMM who use stimulants into the PrEP care continuum. Although some studies found that SMM who use substances report concerns about their hypothetical ability to adhere to PrEP [29, 30], actual PrEP use among SMM who use substances such as methamphetamine and amyl nitrites (i.e., poppers) appears to be comparable to or greater than their peers who do not use substances [31–39]. At the same time, there is growing evidence that SMM who use stimulants and other substances can experience difficulties achieving prevention effective levels of daily oral PrEP adherence [34, 40, 41] that may serve as early indicators of greater risk for disengagement from PrEP care and discontinuing PrEP [42–44]. For example, those engaging in heavy cocaine use have nearly three-fold greater odds of disengagement from PrEP care compared to non-users [42]. Taken together, there is a clear need for scalable interventions to promote entry or re-entry of SMM who use stimulants into the PrEP care continuum.

The effectiveness of motivational interviewing (MI) and contingency management (CM) is supported by decades of clinical research in people with substance use disorders [45], and these are the only interventions that are efficacious for reducing both substance use and CAS in SMM [46–50]. MI is an evidence-based counseling intervention targeting *intrinsic motivation* for health behavior change [51]. CM targets *extrinsic motivation* by providing tangible incentives as positive reinforcement for performing health behaviors [52], and it has also been successfully utilized to promote HIV-related health behavior change in people who use substances [53–58]. Interestingly, findings from one meta-analysis of the substance use disorder treatment literature indicate that MI achieves small but durable outcomes while CM leads to moderate short-term outcomes [59]. PrEP Readiness Interventions for Supporting Motivation (PRISM) addresses an important gap by testing adapted MI and CM interventions for promoting entry or re-entry into the PrEP care continuum.

Cognitive Evaluation Theory proposes that motivational processes are arranged hierarchically such that the extrinsic rewards for behavior change provided during CM could paradoxically undermine intrinsic motivation in some circumstances [60]. In Cognitive Evaluation Theory, higher order needs for autonomy and self-determination govern the regulation of intrinsic motivation, both of which could be partially undermined by CM incentives. This underscores the need for clinical research to test if there are distinct combinations of MI and CM that optimize entry or re-entry into the PrEP care continuum among SMM who use stimulants.

**Figure 1:**
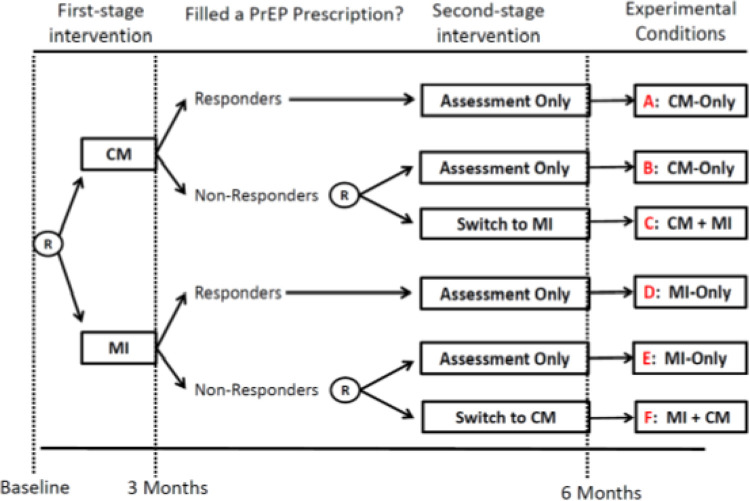
Pilot SMART of the telehealth PRISM motivational enhancement interventions to facilitate entry or re-entry into the PrEP care continuum

This is a protocol for a pilot sequential assignment randomized trial (SMART) testing the feasibility, acceptability, and preliminary effectiveness of the telehealth PRISM motivational enhancement interventions. The primary outcome is documented evidence of filling a PrEP prescription over six months. As shown in Fig. 1, employing sequential randomization procedures allows for a comparison of two first-stage interventions: MI versus CM. Then, among those who do not report filling a PrEP prescription after three months (i.e., non-responders) we conducted a second randomization to: *Switch* to a second-stage intervention (i.e., MI + CM, CM + MI) versus *Continue* with assessments only. Because CM has been shown to yield moderate short-term effects, we hypothesize that a greater proportion of those randomized to receive CM as the first-stage intervention will have filled a PrEP prescription and report being evaluated for PrEP by a medical provider over six months. Because MI targets concomitant HIV risk behaviors, we hypothesize that those randomized to receive it as the first-stage intervention will show greater reductions in self-reported stimulant use and CAS over six months. Finally, among non-responders we hypothesize that participants randomized to *Switch* to a second-stage intervention (i.e., CM + MI, MI + CM) will have greater improvements in the primary and secondary outcomes compared to those who *Continue* with assessments only.

## Method And Design

This pilot SMART randomized 70 HIV-negative, SMM who use stimulants (i.e., methamphetamine, powder cocaine, or crack-cocaine) that are not currently taking PrEP and trial completion is anticipated in June of 2023. Participants were recruited via social networking applications (e.g., Grindr, Scruff) across the United States. Interested individuals are directed to an online screener that assesses the following inclusion criteria: 1) 18 years of age or older; 2) identifies as a cisgender man; 3) reports anal sex with a man (past 6 months); 4) reports using methamphetamine, powder cocaine, or crack-cocaine in the past 3 months; 5) HIV-negative or unknown serostatus; 6) meets CDC criteria for PrEP eligibility [61]; and 7) not currently prescribed PrEP. PrEP eligibility was operationalized as: 1) any CAS regardless of partner type; or 2) any sexually transmitted infection (past six months). All procedures for this pilot SMART were approved by the University of Miami Institutional Review Board with a reliance agreement from the City University of New York Graduate School of Public Health.

All pilot SMART activities were conducted using HIPAA-compliant Zoom, which emerged as a flexible response to the COVID-19 pandemic and represents a scalable platform for reaching the broader population of SMM who use stimulants with the telehealth PRISM motivational enhancement interventions. Potentially eligible participants were invited to an enrollment visit that included an informed consent for the first three months of the pilot SMART and a baseline assessment. Those completing the enrollment visit received a $50 Amazon gift card, Venmo, or Zelle cash application payment.

### Mail-in HIV testing.

After the baseline assessment, participants are sent a testing kit to collect a saliva sample with a OraSure HIV-1 (LOT 6691354) testing device, and they receive a $25 Amazon gift card or cash application payment for mailing this sample to a laboratory for HIV testing [62, 63]. Participants who did not complete HIV home testing or those have reactive HIV test results are excluded. Participants with reactive test results are provided with post-test counseling and assisted in connecting with local confirmatory testing [64].

### Sequential informed consent.

At baseline, participants provide informed consent for the first three months of the pilot SMART with trained intervention staff. This consent describes the first-stage randomization to MI versus CM as well as the 3-month follow-up assessment. We employed sequential informed consent procedures to ensure that participants do not plan to delay filling a PrEP prescription to be eligible for a second-stage randomization and the possibility of additional incentives (e.g., those initially randomized to MI would delay filling a PrEP prescription to have another chance to receive CM financial incentives). After the 3-month follow-up assessment, participants complete a second informed consent that describes procedures for the second-stage randomization of non-responders and the 6-month follow-up assessment for all participants. The follow-up assessments at three and six months are completed by an independent assessor who has not previously delivered MI or CM to participants. Participants are sent a link to the self-report measures to complete on their own and they receive a $50 Amazon gift card or cash application payment for completing each assessment.

### Pilot SMART procedures.

As shown in Fig. 1, participants who provide a test that is non-reactive for HIV are randomized to receive a first-stage intervention: 1) a 2-session MI intervention; or 2) CM where they receive up to $100 in financial incentives for taking the first steps towards (re-)starting PrEP. Randomization at baseline is stratified as a function of whether participants have previously taken PrEP with the randomization schemes created in SAS v. 9.4 by the statistician/data manager using randomly permuted block sizes of two, four, and six. The randomization schemes were administered using REDCap and were not viewable by staff until the moment of randomization for each participant. Immediately following randomization, participants receive their first MI session or a brief introduction to the financial incentives they can receive during the 3-month CM period. All participants receive a $20 Amazon gift card or cash application payment for attending this first-stage randomization visit. After three months, participants who report that they have not filled a PrEP prescription in the prior three months are classified as non-responders. Non-responders are randomized a second time (no stratification) to either: 1) *Switch* to a second-stage intervention (MI + CM or CM + MI); or 2) *Continue* with assessments only.

Finally, both responders and non-responders complete a final follow-up assessment at six months to measure the primary and secondary outcomes.

### MI intervention.

The adapted, 2-session MI intervention simultaneously targets PrEP use (session one) as well as co-occurring substance use and CAS (session two). Each session is delivered approximately one week apart, and participants receive a $20 Amazon gift card or cash application payment at each session. However, participants have up to three months to complete both MI sessions.

Session one focuses extensively on enhancing intrinsic motivation for (re-)starting PrEP. To begin, participants are invited to tell the facilitator a bit about themselves and describe what they know about PrEP. Using *elicit-provide-elicit (EPE) techniques*, the facilitator provides relevant information to address any gaps in knowledge about PrEP. This can include topics like the effectiveness and safety of PrEP, prevention effective adherence levels for SMM, different oral dosing strategies (e.g., 2-1-1), navigating PrEP care, and the availability of financial support for those without insurance. Then, participants are invited to describe their thoughts and feelings about the possibility of starting PrEP with selective reinforcement of change talk by the facilitator. Using the *change ruler*, participants rate on a scale of one to ten how important it is for them to see a medical provider to learn more about PrEP. The facilitator uses this to elicit change talk by asking participants why they did not pick a lower number (e.g., “Why did you pick a three and not a one?”). Next, the facilitator engages participants in the *roadmap exercise* where the decision regarding whether to start PrEP is presented as a fork in the road. Participants are asked to consider what life might look like in 1–2 years if they did not start PrEP and then if they chose to start PrEP. The session ends with a summary and participants are encouraged to examine what (if any) steps they are ready to take toward (re-)starting PrEP. Those who are actively planning to (re-)start PrEP are asked to elaborate on the timeline and examine any potential barriers they might encounter in accessing PrEP care.

At the beginning of session two, participants are asked to describe their current thoughts and feelings about (re-)starting PrEP. The facilitator selectively reinforces change talk and answers any questions participants might have about navigating PrEP care. Next, participants are introduced to the focus of session two, concomitant stimulant use and CAS. Participants are presented with a menu of options that reflect possible topics for the session including: 1) change how I use stimulants; 2) start substance use treatment; 3) attend a self-help group; 4) abstain from stimulants; 5) fewer sex partners; 6) less sex on stimulants; 7) use condoms; and 8) get tested for HIV regularly. Participants are encouraged to identify a topic of focus for the session that he would like to discuss at greater length. Our approach to addressing stimulant use and CAS embraces the philosophy of harm reduction such that participants are not required to be ready, willing, or able to abstain from stimulant use or use condoms every time during anal sex [65]. Instead, the facilitator assists participants with exploring a range of possible behavior change targets such as reducing the frequency of stimulant use, changing the mode of stimulant administration (e.g., snorting instead of smoking), and using condoms during receptive anal sex with casual partners. Once a behavior change target is identified, participants are encouraged to elaborate on it to reinforce change talk. Using the *change ruler*, participants rate on a scale of one to ten how important it is for them to make this change. The facilitator uses this to elicit change talk by asking participants why they did not pick a lower number (e.g., “Why did you pick a three and not a one?”). Next, the facilitator engages the participant in the *roadmap exercise* where the decision regarding whether to make this change is presented as a fork in the road. Participants are asked to consider what life might look like in 1–2 years if they did not make a change and then if they chose to change. The session ends with a summary and participants are encouraged to examine what steps (if any) they are ready to take towards change. Those who are actively planning to make a change in their behavior are asked to elaborate on the timeline and examine any potential barriers they might encounter.

### CM intervention.

Facilitators provide a brief overview of the CM protocol and encourage participants to ask any questions they may have about how to access PrEP in their community. Facilitators provide tailored referrals for local PrEP services (e.g., www.preplocator.org) and online PrEP providers. Participants are provided financial incentives as positive reinforcement for two key behaviors that are crucial to PrEP care continuum (re-)entry. First, participants can receive a $50 incentive for documented evidence of completing a medical visit for PrEP clinical evaluation that includes HIV testing. Documentation that the participant can include either a note from a medical provider indicating that the participant was seen for a PrEP evaluation or laboratory results consistent with PrEP clinical evaluation (e.g., HIV testing, kidney function). Second, participants who provide evidence that they have filled a recent PrEP prescription for Truvada or Descovy matched to their photo identification (e.g., photo of the medication bottle), will receive an additional $50 incentive. Participants on Aperatude as injectable PrEP are asked to provided documentation from their provider or medical record demonstrating their received an injection to obtain the $50 incentive. Because filling a PrEP prescription requires participants to receive PrEP clinical evaluation by a medical provider, those who have not previously received $50 for PrEP clinical evaluation receive $100 when they provide evidence they have filled a PrEP prescription. In order to provide timely positive reinforcement for these behaviors, incentives are provided via remote payment applications (e.g., Venmo, Zelle) within two business days.

### Fidelity monitoring.

The procedures for monitoring the fidelity of the CM and MI intervention protocols are as follows: 1) detailed curriculum manuals for each intervention; 2) intensive training in delivering MI and CM; 3) incorporation of mock training sessions to ensure staff meet performance criteria for delivery; 4) facilitator checklists; and 5) audio taping of sessions. Audio recordings of facilitator sessions are reviewed to assess adherence to the protocol, delivery, interpersonal skills, facilitator/participant rapport, and session flow. Fidelity monitoring is also crucial to mitigate the risk of contamination (e.g., possible delivery of MI counseling during CM). The first three participants for each staff member (MI and CM) are reviewed immediately. This is followed ongoing individual and group supervision. Fidelity ratings of MI sessions use the MI treatment integrity (MITI) protocol [66], which is the gold standard approach for monitoring the fidelity of MI. MITI scores review four global categories (cultivating change talk, softening sustain talk, partnership and empathy), and are based on a Likert-scale from one (low) to five (high) [67]. To date, our team has completed 77 MI sessions conducted by 4 facilitators and all sessions have been rated using the MITI. Mean global scores included: cultivating change talk mean 4.36 (SD- 0.6); softening sustain talk mean 3.91 (0.51); partnership 4.46 (0.57) and empathy mean 4.24 (0.59).

### Primary outcome.

The primary outcome is verified evidence that a participant has filled a PrEP prescription (i.e., photo of a recently filled medication bottle) over the 6-month follow-up. For participants who do not provide evidence of a PrEP prescription as part of CM, those who report taking PrEP are subsequently notified that they can receive an additional $20 incentive for providing evidence of a recent PrEP prescription. We chose to implement this strategy to mitigate differential assessment of the primary outcome due to CM incentives. Participants are offered the $20 incentive for providing evidence only after they self-report recently filling a PrEP prescription so that it does not function as a CM incentive.

### Secondary outcomes.

Participants complete self-report measures over the 6-month follow-up to assess several secondary outcomes that are described briefly below.

#### PrEP clinical evaluation.

Participants are asked to indicate if they attended an appointment with a medical provider about starting PrEP in the past three months. Those who indicate they attended any medical appointment about starting PrEP over the 6-month follow-up are classified as completing PrEP clinical evaluation.

#### Stimulant use severity.

The World Health Organization Alcohol, Smoking, and Substance Involvement Screening Test (ASSIST) is a validated self-report measure of substance use [68, 69]. We will examine changes in the amphetamine-type stimulant and cocaine use composite scores, which measure of the severity of symptoms for stimulant use disorders. Where participants are using multiple stimulants at a given time point, we will select the highest mean score. We will also examine changes in clinically validated cut-points for moderate and severe stimulant use disorders using the ASSIST (Mild = 0–3; Moderate = 4–26; Severe = 27 or greater). Again, where participants are using multiple stimulants at a given time point, we will select the cocaine or amphetamine-type stimulant use score that reflects higher severity.

#### Receptive and insertive CAS.

Participants are asked to separately indicate the number of men with whom they had receptive or insertive anal sex “without a condom at least part of the time” in the past three months. Where participants report CAS partners, they are asked to separately indicate the number of receptive and insertive CAS partners who were taking PrEP or were HIV + undetectable to examine the extent to which participants are biomed sorting [70].

#### PrEP intentions.

Based on the Information-Motivation-Behavioral Skills (IMB) model for understanding PrEP intentions [71], this three-item PrEP intentions subscale assesses the participant’s intentions to complete certain PrEP-related behaviors within the next three months (e.g. “During the next three months, I will talk to a health care provider about PrEP.”) Responses on a four-point scale range from Definitely Will Not Do (1) to Definitely Will (4) with higher scores indicating stronger intentions to use PrEP.

#### PrEP self-efficacy.

Based on the IMB model, this eight-item scale assesses how difficult participant’s view behavior skills related to PrEP use (e.g., “How difficult would it be for you to talk openly and honestly with a doctor about your sexual behaviors?”). Participant response options on a four-point scale range from Very Hard to Do (1) to Very Easy to Do (4) with higher rankings indicating greater self-efficacy for PrEP use [71].

#### PrEP attitudes.

A subset of the IMB PrEP intentions model [71], this five item scale assesses attitudes towards PrEP use (e.g., “PrEP is effective at preventing HIV.”) [71]. Participant response options on a five-point scale range from Strongly Disagree (1) to Strongly Agree (5) with higher scores indicating more positive attitudes towards PrEP use.

#### PrEP stigma.

This five-item scale assesses stigmatizing notions around PrEP use (e.g., “People who take PrEP are promiscuous.”) [71]. Participants response options on a five-point scale range from Strongly Disagree (1) to Strongly Agree (5) with higher scores indicating more stigmatizing views about PrEP [71].

### Intent-to-treat analyses.

Using logistic regression, we will examine the intent-to-treat effects of first-stage randomization (i.e., MI versus CM) on: 1) any documented evidence of filling a PrEP prescription (primary outcome) over the 6-month follow-up; and 2) any self-reported PrEP clinical evaluation by a medical provider over the 6-month follow-up. These logistic regression analyses will test our primary hypothesis that it is better to start with CM versus MI. Intent-to-treat analyses of secondary outcomes involving longitudinal trajectories of continuous measures will be tested using multilevel random coefficient models (i.e., hierarchical linear modeling). These analyses will test our secondary hypotheses that those randomized to receive MI as a first-stage intervention will display greater decreases in stimulant use severity and number of CAS partners over six months. Finally, exploratory analyses using the methods described above will be conducted among non-responders only. We hypothesize that non-responders randomized to *Switch* to a second-stage intervention (i.e., CM + MI, MI + CM) will have greater improvements in the primary and secondary outcomes compared to those who *Continue* with assessments only.

### Power analysis.

Recent statistical modeling clearly demonstrates that it is inappropriate to use effect size estimates from a pilot trial for establishing effectiveness or informing subsequent power analyses [72]. Thus, the most appropriate focus of a pilot is to examine issues relevant to feasibility and acceptability, which does not require adequate statistical power. Assuming N = 70, α = 0.05 (two-tailed), power = 0.80, and 75% retention at six months, minimum detectable standardized mean differences for continuous outcomes ranged from 0.53 to 0.79 for within-subjects correlations r ranging from 0.10 to 0.80. For binary outcomes, using the same inputs as above plus small, medium, and large base rates of 10%, 25%, and 50%, respectively, raw proportion differences for r = 0.10 ranged from 21–25% (standardized difference = .55); for r = 0.80, the corresponding raw proportion differences ranged from 34–38% (standardized difference = 0.80). Although formal hypothesis testing will not be the primary focus, there will be sufficient statistical power to detect medium to large effects for the primary hypothesis comparing whether it is better to start with CM versus MI. Statistical power estimates following the second-stage randomization are dependent on the non-response rate. Estimating this non-response rate will guide a planned hybrid type 1 randomized controlled trial testing the effectiveness of these telehealth PRISM motivational enhancement interventions for promoting (re-)entry of SMM who use methamphetamine into the PrEP care continuum.

### Qualitative exit interviews.

After the 6-month follow-up assessment, participants are purposively selected for an in-depth qualitative interview examining their experiences with the telehealth PRISM motivational enhancement intervention(s) received. Participants are purposively selected for in-depth qualitative interviews based upon if they were responders (n = 15) or non-responders (n = 15) after six months. Participants invited to complete an in-depth qualitative interview receive a $50 Amazon gift card or cash application payment.

Interviews will be conducted by a member of the study team that has not previously interacted with the participants at any point in the trial. Interviews will last approximately 60 minutes and participants will receive a $50 Amazon, Venmo, or Zelle payment for their participation. The interviewer will receive ongoing training and supervision in qualitative methods and the study objectives. The semi-structured interview guide will be tailored based on the participants PrEP status (responder at Stage 1, responder at Stage 2, or non-responder at 6 months). Topics include: likes and dislikes of PRISM interventions components, views on adequacy of study incentives, thoughts about PrEP, interest in injectable PrEP, and barriers and facilitators to PrEP uptake and adherence.

### Qualitative analyses.

Participants interviews will be audio recorded on zoom with the participants consent and transcribed verbatim. The transcripts will be reviewed for accuracy and quality assurance. A general inductive approach [73] will be used to analyze themes relating to the acceptability and feasibility of the PRISM interventions. Codes will be derived inductively starting with a thorough reading of the transcripts and identification of relevant information expressed by participants. An initial codebook will be developed by the primary data analyst after a comprehensive reading of all transcripts. Next, a random sample of five interviews will be selected for a second data analyst to code for inter-rater agreement. The two researchers will review and discuss any discrepancies in the coding, emergent themes, and the need for refined definitions. The researchers will accept all codes where there is agreement and come to a consensus for areas of non-agreement based on their discussions; they will revise the codebook, and re-code the transcripts based on the revised codebook. Next, a second random sample of five interviews will be selected to determine inter-rater agreement. When the analysts reach an inter-rater agreement of 85–90% in the second round of coding the transcript codes will be finalized [74]. Agreement is defined as whether identical codes are applied to the selected text by both coders. Once inter-rater agreement is reached, all interviews will be coded by either one of the 2 analysts based on the final codebook following established guidelines for determining coding saturation [75].

## Discussion

**Figure 2:**
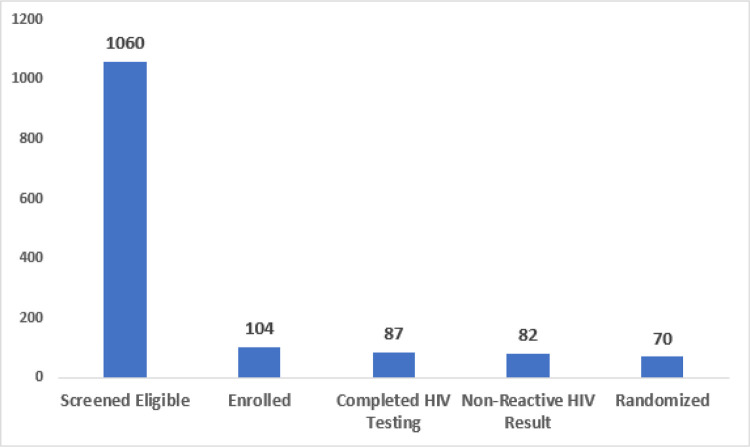
Continuum of engagement for SMM who use stimulants in the pilot SMART of the telehealth PRISM motivational enhancement interventions.

Implementation of the pilot SMART of the PRISM telehealth motivational enhancement interventions highlights the challenges associated with launching and sustaining a national recruitment campaign to achieve our target sample size. As shown in Fig. 2, recruitment of via social networking applications (i.e., Grindr) has proven to be a viable method for reaching a large pool of potentially eligible participants. Although only 14% of those who completed the online screener were eligible, the large number of screeners completed yielded 1,060 potentially participants. However, enrolling potentially eligible participants proved to be more difficult than anticipated. A total of 104 (10%) completed a Zoom enrollment visit and 70 (7%) were randomized in the pilot SMART. This reflects the challenges in optimizing HIV prevention with SMM who use stimulants, a high priority population that is often difficult to access because many are not actively seeking services. Future randomized controlled trials will likely require a substantial investment of time and resources for national recruitment campaigns to examine the effectiveness of the PRISM telehealth motivational enhancement interventions.

Throughout the pilot SMART, we developed and refined strategies to support engagement of potentially eligible participants through text messages and other communications that emphasized the non-judgmental, flexible, and participant-centered approach of our team. We devoted substantial resources to enrolling participants with multiple contacts to facilitate scheduling and often multiple missed enrollment visits. Most participants did not respond to multiple attempts to contact, and staff reported approximately a two-thirds no show rate for Zoom enrollment visits. PRISM staff implemented procedures regarding appointment reminders that included non-judgmental language regarding follow-ups for missed appointments (e.g., “Sorry we missed each other today. Happy to reschedule for another time.”) and encouraged participants to re-establish contact. This is consistent with findings from previous studies using social media to recruit SMM most vulnerable to HIV acquisition [76, 77] as well as text messages to enhance engagement and retention [78].

Despite the challenges we experienced in enrolling potentially eligible participants, there were no significant differences between enrolled and non-enrolled groups in race/ethnicity, age, and type of stimulant(s) used. As shown in Table 1, 44% of enrolled participants were ethnic minorities with an average age of 38.5 (SD = 8.9) years. Most participants reported only methamphetamine use in the last three months (63%), followed by co-use of methamphetamine and powder/crack cocaine (22%), and then only powder or crack cocaine use in the last three months (15%). Although enrolled participants were more likely to know about “on demand PrEP” or “event-based dosing” as an alternative to daily oral PrEP, nearly three-fourths of enrolled and non-enrolled participants expressed interest in this dosing strategy. Taken together, enrolled participants appear to be generally representative of the broader population of SMM who use stimulants that we screened from social networking applications.

The inclusion of HIV testing provided additional successes and challenges for our staff. Staff time was required to assemble and mail HIV testing kits, resending kits as needed, as well as time spent texting participants reminders to return their saliva specimens. Throughout implementation of the pilot SMART, we refined standard operating procedures for ensuring timely completion of mail-in HIV testing and delivery of reactive HIV results. As shown in Fig. 1, 87 of the 104 enrolled participants (84%) provided a saliva sample for mail-in HIV testing. Notably, we identified five new HIV infections (6%) and these men were provided with post-test counseling as well as referrals to confirmatory testing. Although mail-in HIV testing with a $25 incentive was feasible and acceptable in this pilot SMART, conducting rapid HIV self-testing during the enrollment visit would have removed operational barriers to completing HIV testing as a prerequisite for randomization. There were routine delays of approximately six weeks in receiving HIV test results for the laboratory. Because non-reactive HIV results were required prior to the randomization visit, these delays may have contributed to attrition of some otherwise eligible participants during this waiting period.

With the onset of the COVID-19 pandemic in 2020, our team revised the pilot SMART protocol to focus on telehealth delivery of MI and CM to national sample. Instead of in-person, local sessions, we were able to enroll SMM who use stimulants from over 30 states. The feasibility of the telehealth approach is supported by high rates of engagement in the MI and CM interventions. Of the 82 with non-reactive HIV results, 70 (85%) attended an incentivized randomization visit where they received either their first MI session or an overview of the CM protocol. Across the first-stage and second-stage intervention periods, we successfully delivered 75 of 78 MI sessions (96%) and all CM randomized to CM received an overview of the available financial incentives. Our findings mirror other studies that found favorable responses to use of telehealth in research settings [79, 80].

Recruiting a national sample also expanded the reach of the pilot SMART to test the potential benefits of delivering the telehealth motivational interventions in geographic regions with varying degrees of structural stigma and access to PrEP clinical services. As some of our participants were located in rural areas, many did not have a PrEP provider available within a reasonable traveling distance. This required referrals for online PrEP delivery options that were the only source of PrEP access for some participants. Randomized controlled trials are needed to determine whether and how geographic region moderates the effectiveness of telehealth motivational enhancement interventions with this high priority population.

Development and implementation of the pilot SMART of the PRISM telehealth motivational enhancement interventions for SMM who use stimulants has afforded our team many successes and learning experiences. Our focus on adaptation of the MI and CM intervention protocols for telehealth delivering during COVID-19 pandemic expanded their reach and potential public health impact. We also gained experiences with implementing a national recruitment campaign that will guide appropriate resource allocation in subsequent randomized controlled trials. Implementing a waiting period prior to randomization did not meaningfully diminish our randomization rate and highlighted operational efficiencies leveraging rapid HIV testing that could be employed in subsequent randomized controlled trials. High rates of engagement in the telehealth MI and CM visits provide strong support for the feasibility. Subsequent dissemination efforts will focus on examining multi-level determinants of substance use and CAS in this high priority population using baseline quantitative data. We also anticipate dissemination of intent-to-treat analyses of primary and secondary outcomes from the pilot SMART with findings from qualitative exit interviews in the coming year.

## Figures and Tables

**Table 1:** Comparison of eligible participants who enrolled versus those who did not enroll (N = 1,060)

	Enrolled (n = 104)	Not Enrolled (n = 956)	p-value
	M (SD)	M (SD)	
**Age**	38.5 (89)	38.1 (10.1)	0.69
	n (%)	n (%)	
**Stimulant Use (Past 3 Months)**	16 (15.4)	141 (14.8)	0.76
Powder/Crack Cocaine Only	65 (62.5)	630 (65.9)	
Methamphetamine Only	23 (22.1)	185 (19.4)	
Powder/Crack Cocaine and Methamphetamine			
**Interested in on-demand PrEP**	77 (74.0)	714 (74.7)	0.89
**On-demand PrEP is a good prevention choice for me**	74 (71.2)	671 (70.3)	0.85
**It would be difficult for me to use on-demand PrEP**	9 (8.7)	101 (10.6)	0.54
**Race/ethnicity**	16 (15.4)	76 (8.1)	0.08
Black/African American	58 (55.8)	548 (58.1)	
White	21 (20.2)	212 (22.5)	
Hispanic/Latino	9 (8.7)	108 (11.4)	
Other ethnic minority			
**HIV status**	84 (80.8)	729 (76.3)	0.30
HIV-negative	20 (19.2)	227 (23.7)	
Unknown			
**Diagnosed with a STI (Past 6 Months)**	17 (16.4)	114 (12.0)	0.20
**Any sex exchange for money or drugs**	29 (28.4)	303 (31.9)	0.47
**Aware of on-demand PrEP**	73 (70.2)	575 (60.2)	0.047
**Previously used on-demand PrEP**	4 (3.9)	25 (2.6)	0.52^†^

Note: Chi-squared test of proportions was used for all categorical characteristics except for where denoted by †, where Fisher’s exact test was used. A pooled t-test was used to compare means across both groups for the continuous characteristic, age.

## Data Availability

The datasets used and/or analyzed during the current study are available from the corresponding author on reasonable request.

## Abbreviations

CAS: Condomless anal sex
CDC: US Center for Disease Control and Prevention
CM: Contingency management
EPE: elicit-provide-elicit
MI: Motivational interviewing
PrEP: HIV pre-exposure prophylaxis
PRISM: PrEP Readiness Interventions for Supporting Motivation
MITI: MI treatment integrity
SMART: Sequential multiple assignment randomized trial
SMM: Sexual minority men
